# FeSiCr Alloy Powder to Carbonyl Iron Powder Mixing Ratio Effects on the Magnetic Properties of the Iron-Based Alloy Powder Cores Prepared Using Screen Printing

**DOI:** 10.3390/ma14041034

**Published:** 2021-02-22

**Authors:** Hsing-I. Hsiang, Kai-H. Chuang, Wen-H. Lee

**Affiliations:** 1 Department of Resources Engineering, National Cheng Kung University, Tainan 70101, Taiwan; cathy12345566@gmail.com; 2 Department of Electrical Engineering, National Cheng Kung University, Tainan 70101, Taiwan; leewen@mail.ncku.edu.tw

**Keywords:** magnetic pastes, screen printing, iron-based alloy powder, coils

## Abstract

A screen printing process was used to substitute dry molding to solve the uneven compaction problem in the coil center column during molding in this study. FeSiCr alloy powders (FSC) with a large particle size were mixed with fine spherical carbonyl iron powder to increase the compaction density. FSC to carbonyl iron powder (CIP) mixing ratio effects on magnetic paste rheological behaviors and magnetic properties of the molding coil prepared using screen printing were investigated. A magnetic paste with the lowest viscosity can be obtained using 3C7F (30% CIP + 70% FSC) due to the small-sized CIP adsorbed onto the FSC surface. This process reduces the interlocked network formation resulting from the CIP. The toroidal core with 3C7F exhibited the highest relative density and highest inductance. The coils with pure CIP and higher CIP content exhibited the better DC superposition characteristic. The toroidal core loss increased rapidly as the FSC content was increased. A proper trade-off between the inductance, DC-bias superposition characteristic, and magnetic core loss can be reached by choosing a suitable powder mixing ratio.

## 1. Introduction

The dry molding of alloy magnetic powders directly onto enameled wire is used to prepare the molding coils. The molding coils exhibit smaller dimension, lower profile, and better magnetic properties compared to the wound-type power inductors. Therefore, the molding coils have been used for the power supply of electronic equipment due to the superior magnetic properties [1,2,3]. The small space in the coil center column cannot be well filled with the granules composing of alloy magnetic powder and organic binder during molding the miniaturized coil. This leads to fissures or a porous microstructure in the coil center column. Therefore, how to overcome the uneven compaction of the miniaturized molding coil manufacturing problem becomes an important issue.

Screen-printing technology has been extensively used to mass produce the surface-mounted components. In this study the molding coils were fabricated by using a screen printing process of high-solid-content iron-based alloy magnetic paste. High-solid-content magnetic paste is prepared by mixing solvent, epoxy resin, and iron-based alloy powders. The magnetic paste is screen-printed into a mold with enameled wires and then dried, compacted, and hot-cured to fabricate a coil, which can effectively solve the uneven compaction problem in the coil center column.

FeSiCr alloy alloys (FSC) present better DC-bias superposition characteristics than NiCuZn ferrites [4,5,6,7] and are hence widely used in power molding coils [8,9,10,11]. Moreover, a Cr-rich oxidation layer formed on the FSC surface can increase electrical resistivity to reduce the eddy current loss and improve the oxidation and corrosion resistance of the inductors [12,13]. However, FSC exhibit high hardness and brittleness leading to difficulties to consolidate to full density. Carbonyl iron powder (CIP) exhibits many advantages, such as low cost, high saturation magnetization, and low coercivity [14,15,16]. CIP is also extensively used in molding coils [17]. The compaction density of the molding coils can be enhanced by adding CIP into FSC owing to its large plastic deformation during pressing and can hence increase the inductance. On the other hand, the packing density can also be significantly increased by adding small spherical particles to fill the interstices between the large particles. Therefore, the FSC with a large particle size were mixed with fine spherical carbonyl iron powder to increase the compacted density, and the FSC to CIP mixing ratio on the rheological behaviors of the magnetic pastes and magnetic properties of the molding coils prepared using screen printing were investigated in this study.

## 2. Experimental Procedures

The raw materials and experimental procedures are similar to our earlier studies [18,19] and are described in the Supplementary Materials. The constitution of the magnetic paste is shown in Table 1. The only difference are the magnetic powders with different mixing weight ratios of CIP to FSC (10:0, 7:3, 5:5, 3:7, and 0:10) that were used to prepare the magnetic pastes.

## 3. Results and Discussion

**Figure 1:**
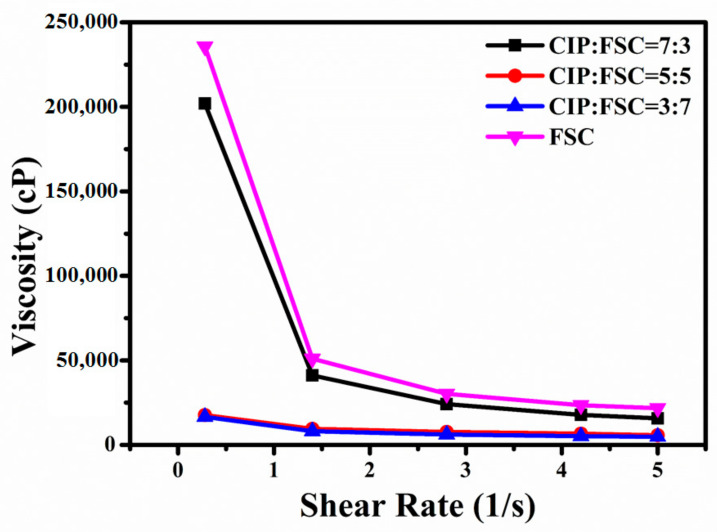
Effect of the different powder mixing ratios on the rheological behaviors of the magnetic pastes.

**Figure 2:**
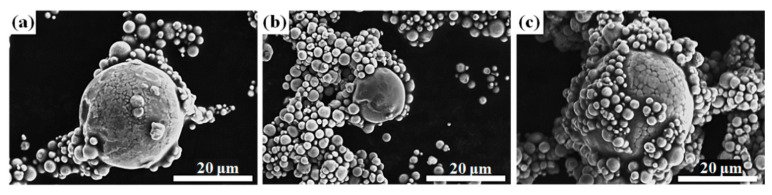
SEM images of the magnetic pastes after drying (**a**) 3C7F, (**b**) 5C5F, (**c**) 7C3F.

Figure 1 shows the effect of different powder mixing ratios on the magnetic paste rheological behaviors. All magnetic paste viscosities decreased with increasing the shear rate, i.e., shear thinning behavior. The magnetic paste rheological behavior with 100% CIP cannot be analyzed because the viscosity is too high. This is because CIP with a small particle size is easy to agglomerate, leading to too high viscosity [20]. The large FSC exhibited strong interparticle magnetic attractive force, leading to higher magnetic paste viscosity with pure FSC. Figure 2 shows scanning electron microscopy (SEM) images of the magnetic pastes after drying. The lowest viscosity was obtained in the 3C7F sample due to the small-sized CIP adsorbed onto the FSC surface. This reduced the interlocked network formation resulting from the CIP (Figure 2a). As the CIP proportion was increased, the CIP did not adsorb onto the increased FSCs, leading to the formation of an interlock network (Figure 2c) and thereby increasing the magnetic paste viscosity.

**Figure 3:**
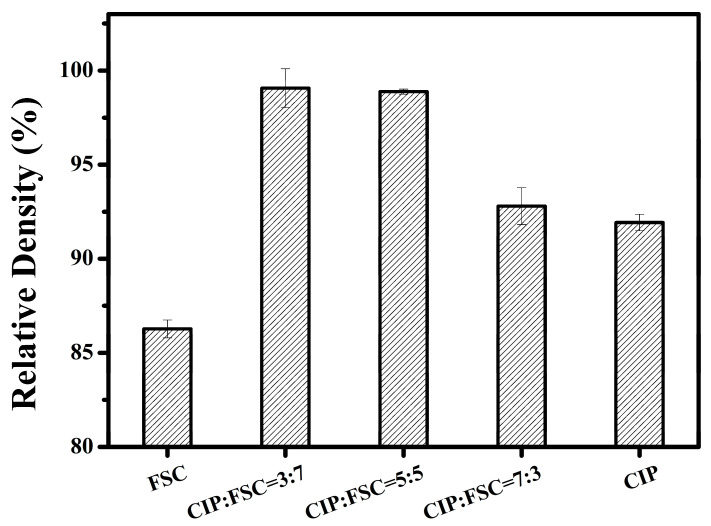
Effect of different powder mixing ratios on the relative toroidal cores densities.

**Figure 4:**
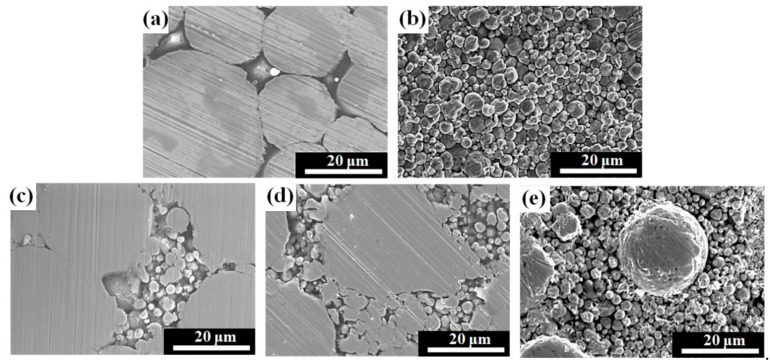
Microstructures of the toroidal cores with different powder mixing ratios (**a**) FSC, (**b**) CIP, (**c**) 3C7F, (**d**) 5C5F, (**e**) 7C3F.

Figure 3 shows the effect of different powder mixing ratios on the relative toroidal core densities. Sample 7F3C exhibited the highest relative density. This is because 30% small-sized CIP can fill the interstices of 70% of the large FSCs, leading to a decrease in porosity (Figure 4c) and increase in the relative density [21]. The relative density decreased with further increasing the CIP content. This is due to the CIP content exceeding the interparticle volume formed by the FSC and widened the FSC interparticle spacing, or the large FSC cannot fill the small interstices formed by the small-sized CIP particles, as shown in Figure 4d,e. The relative CIP density was higher than that of FSC due to the large plastic deformation during compaction.

**Figure 5:**
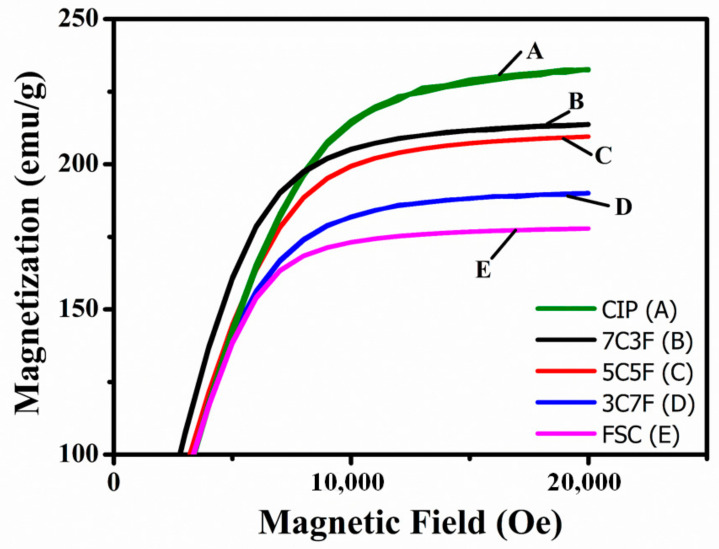
Effect of the different powder mixing ratios on the saturation magnetization of the toroidal cores.

**Figure 6:**
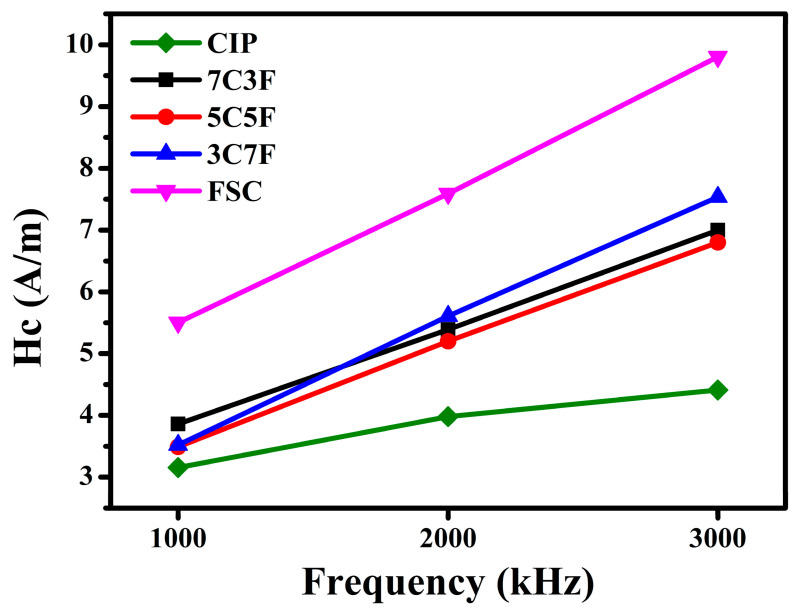
Dependence of the coercivities of the toroidal cores with the different powder mixing ratios.

Figure 5 shows the effect of different powder mixing ratios on toroidal core saturation magnetization. The saturation magnetization is mainly dependent on the constituent phase and relative density [22], because the CIP saturation magnetization is higher than that of FSC [23,24]. The saturation magnetization decreased with increasing the FSC content. The variation of the coercivity in toroidal core with the different powder mixing ratios is shown in Figure 6. As the measurement frequency increased, the coercivity increased due to the eddy current effect for all samples [25]. Compared to FSC, CIP exhibits lower coercivity due to less impurity content 14The coercivities of samples 3C7F, 5C5F, and 7C3F are very close. The coercivity is strongly influenced by the pores [26]. Although 5C5F has a lower CIP content, the coercivity of 5C5F is lower than that of 7C3F due to the higher relative density.

**Figure 7:**
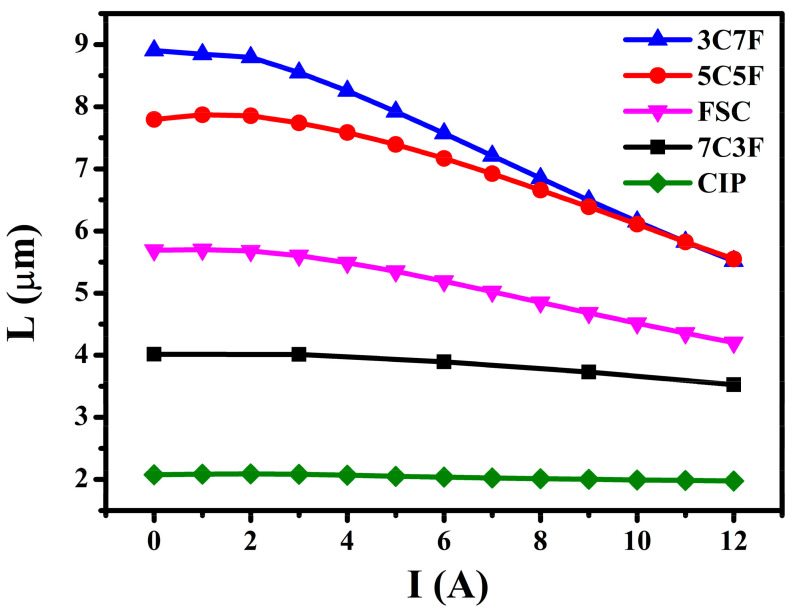
Dependence of the inductance values at 1 MHz with the DC-bias current for the coils.

Figure 7 shows the dependence of the inductance values at 1 MHz with the DC-bias current for the coils (placing enameled wire in a mold). The inductance depends strongly on the relative density and constituent phase. The coil with pure FSC had higher inductance than pure CIP due to the higher FSC permeability. The coil with 3C7F exhibited the highest inductance due to the highest relative density and higher FSC content. On the contrary, the coils with 7C3F had relatively low inductance due to the lower relative density and FSC content.

**Figure 8:**
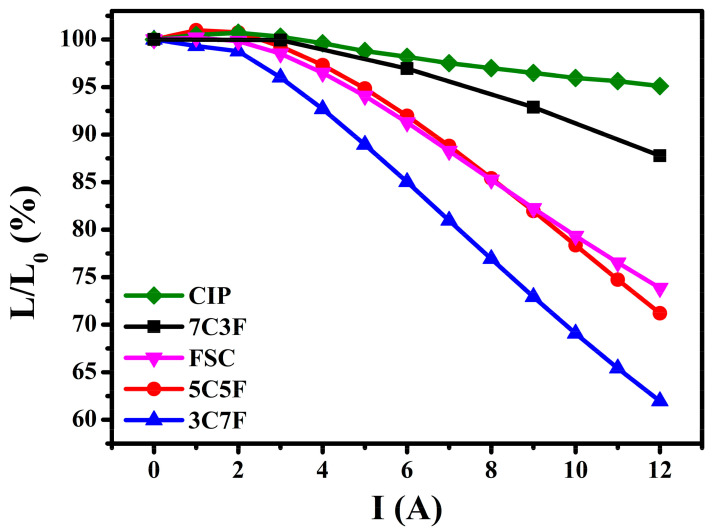
DC-bias superposition characteristics of the coils with different powder mixing ratios.

Figure 8 shows the DC-bias superposition characteristics of coils with different powder mixing ratios. Tang et al. [27] reported that higher saturation magnetization favor better DC-bias superposition characteristics. A magnetic material with a higher saturation magnetization can maintain a linear relationship between magnetization and magnetizing field, resulting in higher incremental permeability at a higher DC current [28]. Therefore, when large current flows through inductors made of a magnetic material with a higher saturation magnetization, the inductance will not suddenly deteriorate. Coils with pure CIP exhibited the best DC superposition characteristic due to the highest saturation magnetization. Coils with 7C3F exhibited the second best DC superposition characteristic due to higher CIP content. Although 3C7F and 5C5F had higher saturation magnetization than FSC, the DC superposition characteristics of 3C7F and 5C5F are poorer than that of FSC. It can be explained that FSC had the higher coercivity, which resulted in a higher demagnetizing field and thereby having more stable incremental permeability under a superposition magnetic field.

**Figure 9:**
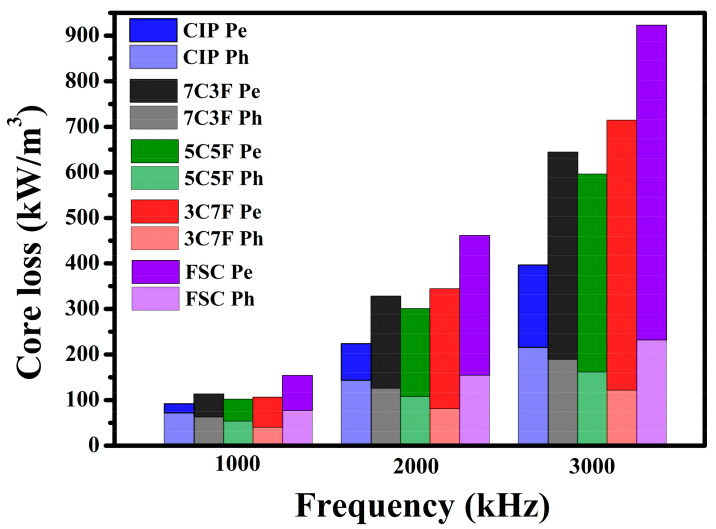
Variation of hysteresis loss (P_h_) and eddy current loss (P_e_) with the measurement frequency for the toroidal cores.

Figure 9 shows the variation of hysteresis loss (P_h_) and eddy current loss (P_e_) with the measurement frequency for the toroidal cores. The magnetic core loss is mainly made up of hysteresis loss and eddy current loss and can be demonstrated as shown in Equation (1) [29].

P_cv_ = P_e_ + P_h_ = K_e_ × f^2^ + K_h_ × f (1)

where K_e_, f, and K_h_ are eddy current loss factor, frequency, and hysteresis loss coefficient, respectively. At low frequency, the magnetic core loss is dominated by the hysteresis loss. The variation in hysteresis loss with different powder mixing ratios has the same trend with the coercivity. The eddy current losses increased rapidly with frequency for all samples. The eddy current loss can be separated into inter-particle eddy current loss and intra-particle eddy current loss [30]. The FSC eddy current loss was much higher than that of CIP, which may be due to the larger particle size resulting in higher intra-particle eddy current loss [31]. Therefore, the toroidal core eddy current loss increased rapidly as the FSC content was increased. Note that the core losses increased rapidly as the frequency was increased above 1 MHz due to the increase in eddy current loss. The size and operating frequency of the converters are limited by the temperature rise resulting from the core loss of molding coils. The core losses of FSC at high frequency can be effectively reduced by mixing with fine spherical CIP and hence widening the operating frequency and minimizing the dimension of the molding coils used in converters.

These results suggest that a suitable trade-off between the inductance, DC-bias superposition characteristic, and magnetic core loss can be reached by choosing a suitable powder mixing ratio.

## 4. Conclusions

1. A magnetic paste with the lowest viscosity can be obtained using 3C7F due to the small-sized CIP adsorbed onto the FSC surface. This reduces in interlocked network formation resulting from the CIP;
2. The toroidal core with 3C7F exhibited the highest relative density because 30% small-sized CIP can fill the interstices of the 70% large FSC, leading to a decrease in the porosity and increase in the relative density;
3. Because the CIP saturation magnetization is higher than that of FSC, the saturation magnetization decreased with increasing the FSC content;
4. The coil with 3C7F exhibited the highest inductance due to the highest relative density and higher FSC content. The coils with pure CIP and higher CIP content (7C3F) exhibited the better DC superposition characteristics;
5. The FSC magnetic core loss was much higher than that of CIP, which may be due to the larger particle size resulting in higher intra-particle eddy current loss. Therefore, the toroidal core loss increased rapidly as the FSC content was increased;
6. A suitable trade-off between the inductance, DC-bias superposition characteristic, and magnetic core loss can be reached by choosing suitable powder mixing ratio.

## Figures and Tables

**Table 1:** Constitution of the magnetic paste.

Magnetic Powder	Binder *	Butyl Carbitol Acetate ** (g)
97.5 wt.%	2.5 wt.%	3.5

*: Epoxy resin: monomer = 8:2 and curing agent: 11.6 phr; **: total weight is 100 g.

## Data Availability

The data presented in this study are available in article.

## Supplementary Materials

The following are available online at https://www.mdpi.com/1996-1944/14/4/1034/s1, Experimental procedure.
