# Supplementary material for: FeSiCr Alloy Powder to Carbonyl Iron Powder Mixing Ratio Effects on the Magnetic Properties of the Iron-Based Alloy Powder Cores Prepared Using Screen Printing
Source: Materials (Basel). 2021 Feb 22;14(4):1034. doi: 10.3390/ma14041034 (PMC7926424; doi:10.3390/ma14041034)
Supplement: Supplementary file 1 [file materials-14-01034-s001.pdf]

materials

MDPI

Supplementary Materials

FeSiCr alloy Powder to Carbonyl Iron Powder Mixing Ratio Effects on the Magnetic Properties of the Iron-Based Alloy Powder Cores Prepared Using Screen Printing

Hsing-I Hsiang [1,*], Kai-Hsin Chuang [1] and Wen-Hsi Lee [2]

1 Department of Resources Engineering, National Cheng Kung University, 70101 Tainan, Taiwan; cathy12345566@gmail.com
2 Department of Electrical Engineering, National Cheng Kung University, 70101 Tainan, Taiwan; leewen@mail.ncku.edu.tw
* Correspondence: hsingi@mail.ncku.edu.tw

1. Materials

Commercial FeSiCr powder (Chung Yo Materials Co. Ltd., Kaoshiung, Taiwan)(D_{50} = 28.7 μm, Fe:Si:Cr = 83.82:14.05:2.13 at%, true density =7.41 g/cm^3) and carbonyl iron powder (Chung Yo Materials Co. Ltd., Kaoshiung, Taiwan)(D_{50} = 3.3 μm, Fe:C = 84.83:15.17 at%, true density =7.74 g/cm^3) was used as the raw material. The standard liquid Bisphenol A epoxy resin (First Chemical, Taipei, Taiwan) with molecular weight of 189 g/mol and butyl glycidyl ether (TCI, Taipei, Taiwan) as the monomer was used. Dicyandiamide (DICY) and 2-methylimidazole (2-MI) purchased from Acros Chemical (Geel, Belgium) were mixed at the ratio of 5/95 and used as the epoxy resin curing agent. Titanate coupling agent, tri(dioctyl) pyro-phosphato titanate-Lica 38 (Kenrich Petrochemicals Inc.,Bayonne, NJUSA), was used without further purification. Butyl carbitol acetate was used as the solvent without further purification.

2. Magnetic Paste Preparation

The paste compositions are shown in Table 1(main text). The epoxy resin, monomer, curing agent, and solvent were mixed by hand. The magnetic powders with different mixing weight ratios of CIP to FSC (10:0, 7:3, 5:5, 3:7, and 0:10) were then added and mixed by planetary mixer for 1 h to prepare the magnetic pastes. The magnetic paste solid content is calculated according to Equation (S1).

$$\text{Solid content} = \frac{\text{powder}}{\text{Epoxy+Monomer+Hardener+Catalyst+powder}}. \quad (1)$$

3. Sample Preparation

The magnetic pastes were screen-printed onto the molds with toroid or coil shapes (placing enameled wire in a mold) and then dried at 100 °C to remove the solvent. The paste was then uniaxially compacted at 900 MPa to form a toroid or coil. The temperature was then increased to 180 °C at a heating rate of 5 °C/min and soaked for 1 h to harden.

4. Characterizations

A viscometer (Brookfield, DV-III ULTRA, Middleboro, MA, USA) was employed to measure the rheological properties of the suspensions at 25 °C. The relative sample density was measured using the Archimedes' method. Scanning electron microscopy (Hitachi, SU1510, Tokyo, Japan) and energy-dispersive spectrometer (BRUKER, XFLASH-Detector 6/10, Berlin, Germany) were employed to observe the microstructure and element

distribution. The hysteresis loop was measured using a superconducting quantum interference device magnetometer (MPMS SQUID VSM, Quantum Design, San Diego, CA, USA) to obtain the saturation magnetization. The initial permeability was measured using an LCR meter, (YHP 4291B, YHP Co., Ltd Hyogo, Tokyo, Japan) with the HP 16454A magnetic material test (YHP Co., Ltd Hyogo, Tokyo ,Japan) fixture from 1 MHz to 1 GHz. The magnetic core loss and coercivity at various measurement frequencies were measured using the B-H analyzer (SY-8218, IWATSU Electronics CO., Ltd, Tokyo,Japan). The incremental permeability under DC-bias superposition condition (DC-bias superposition characteristic) was measured at 1 MHz using a magnetic device analyzer (WK3260B, Wayne Kerr Electronics Co., Ltd, London, UK) with a DC-bias current source (WK3265B, Wayne Kerr Electronics Co., Ltd, London, UK).
